# Carcinoma of Unknown Primary in a Patient With Lynch Syndrome

**DOI:** 10.7759/cureus.15690

**Published:** 2021-06-16

**Authors:** Nat C Jones, Jacob J Adashek, Bassam Ayoub

**Affiliations:** 1 Internal Medicine, University of South Florida (USF) Health, Tampa, USA

**Keywords:** cancer immunotherapy, translational and precision medicine, carcinoma of unknown primary, next-generation sequencing, tissue agnostic approval

## Abstract

Lynch syndrome is the most common form of hereditary colorectal cancer and is associated with an increased incidence of endometrial cancer, small bowel cancer, and other malignancies. The advent of immune checkpoint blockade, next-generation sequencing, and precision medicine molecular tumor boards have revolutionized the current treatment landscape for many cancers and allowed for more creative approaches to treating patients. In addition, tissue agnostic approvals have also served as valid treatment options for patients with otherwise untreatable cancers. In this report, we discuss the case of a 70-year-old woman with Lynch syndrome found to have retroperitoneal lymphadenopathy with p16-negative squamous cell carcinoma, diagnosed as carcinoma of unknown primary (CUP). To our knowledge, this is the first report of Lynch syndrome-associated squamous cell CUP. More research is needed on newly emerging cancer presentations in Lynch syndrome patients as they achieve longer lifespans.

## Introduction

Lynch syndrome is the most common form of hereditary colorectal cancer, accounting for 2-5% of all colorectal cancers [1]. It is also associated with increased incidences of endometrial cancer, small bowel cancer, and several other malignancies [2]. It is characterized by mutations in mismatch repair (MMR) genes, such as MLH1, MSH2, MSH6, and PMS2, which were observed in 13.8% of patients in a colorectal cancer cohort study [3].

There is a paucity of reports describing squamous cell carcinomas (SCC) in patients with Lynch syndrome, with many such reports being on cutaneous SCC [4]. The few instances of Lynch syndrome-associated non-cutaneous SCC that have been reported are thought to be attributed to progressively increasing lifespans of patients with Lynch syndrome who live longer lives with better management of their classically-described cancers, and novel pathologies are now emerging [5].

Carcinoma of unknown primary (CUP) is by default a diagnosis of exclusion. This diagnosis is coined when the histopathology is unclear and a primary tumor cannot be identified. CUPs can have characteristics of adenocarcinomas, undifferentiated carcinomas, and SCCs [6]. Because CUP is a rare diagnosis and treatment generally includes combination chemotherapy without immunotherapy or targeted therapies, patient prognoses are generally poor [7]. The median overall survival (OS) for patients diagnosed with CUP is ~5 months, with improved survival in recent years for some subsets of patients with CUP [8].

In this report, we discuss the case of a patient with Lynch syndrome and a remote history of endometrial and rectal adenocarcinomas and newly diagnosed CUP of squamous origin. To our knowledge, this represents the first reported case of squamous CUP in a patient with Lynch syndrome.

## Case presentation

A 70-year-old woman with a history of Lynch syndrome with Muir-Torre variant [9], who previously had undergone a total abdominal hysterectomy, bilateral salpingo-oophorectomy, and para-aortic lymph node resection with adjuvant radiation in 1997 for stage IIIC endometrial adenocarcinoma and more recently underwent an abdominal perineal resection for stage IIA T3N0M0 rectal adenocarcinoma where immunohistochemistry (IHC) of her rectal mass showed loss of PMS2 and MLH1, with normal MSH2/6, presented with three months of nausea, vomiting, weakness, and 20-pound weight loss.

**Figure 1:**
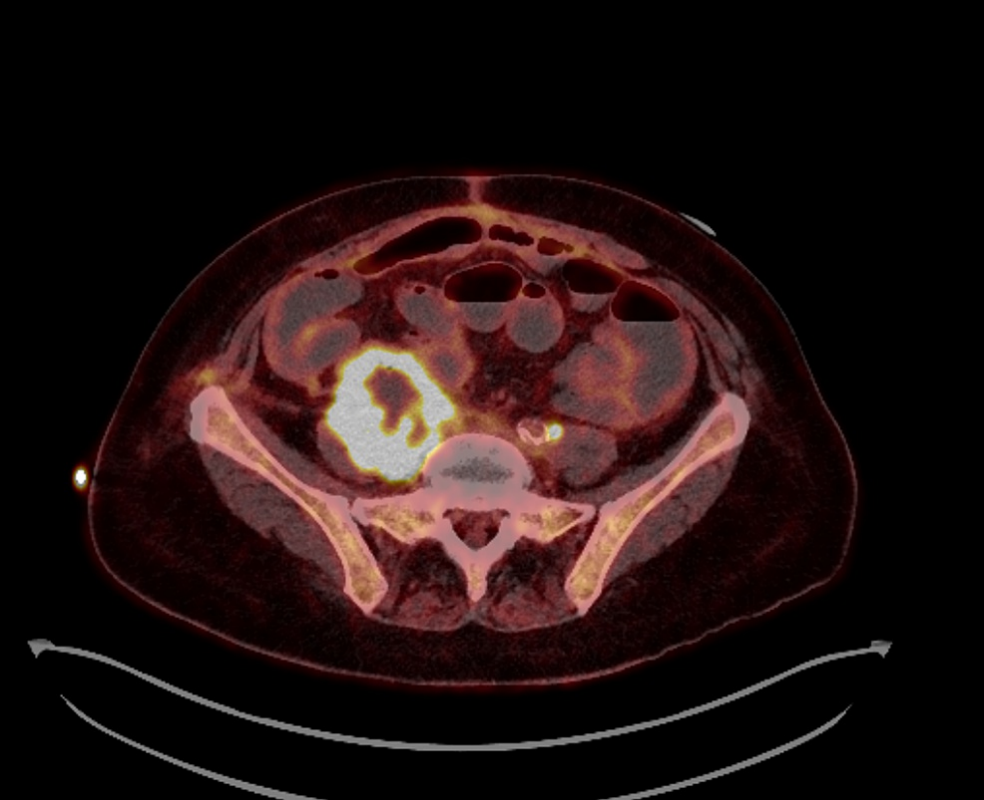
Positron emission tomography scan of the retroperitoneal mass Axial view of the 7.3-cm centrally necrotic retroperitoneal mass is visualized along with a hypermetabolic left para-aortic lymph node.

**Figure 2:**
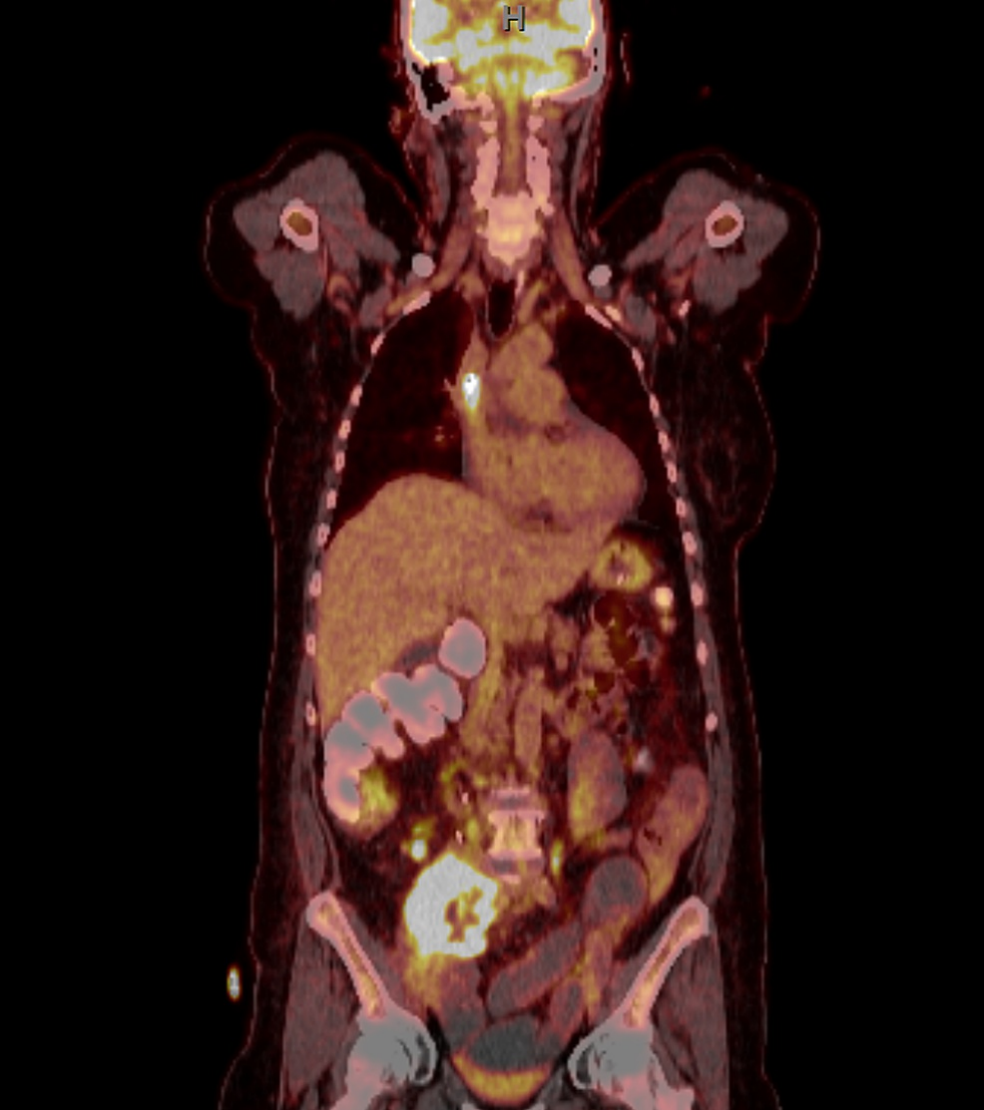
Positron emission tomography scan of the retroperitoneal mass Coronal view of the 7.3-cm centrally necrotic retroperitoneal mass is visualized along with hypermetabolic left supraclavicular, para-aortic, and mesenteric lymph nodes.

She underwent a computed-tomography scan, which revealed a new right-sided 7.3 cm retroperitoneal (RP) mass with additional lymphadenopathy, and underwent a biopsy of the retroperitoneal mass. The pathology revealed squamous histology, negative for p16, inconsistent with her prior adenocarcinomas, making disease recurrence less likely and suggesting a new primary SCC. Endoscopic evaluations of her oropharynx and larynx and both gynecologic/rectal exams failed to identify a primary site of the SCC. A positron emission tomography revealed hypermetabolic left supraclavicular, para-aortic, and mesenteric lymph nodes in addition to the 7.3-cm centrally necrotic RP mass (Figures 1, 2). Thus, the diagnosis of CUP was confirmed. The RP biopsy was sent for next-generation sequencing (NGS), which revealed microsatellite instability-high (MSI-H) and a tumor mutational burden (TMB) of 15 mutations/megabase (mt/mB). Genomic analysis, including a 324 gene panel, revealed mutations in ATM, ARID1A, and KRAS.

The patient was planned to receive pembrolizumab based on having both MSI-H and TMB of 15 mt/mB. Two days prior to the planned time of treatment initiation, the patient acutely decompensated and expired in the intensive care unit.

## Discussion

Precision medicine offers exciting possibilities for the treatment of cancer. Although this patient expired due to an acute complication, her NGS results indicated several reasons for a potential response to immune checkpoint inhibition (ICI). Pembrolizumab is tissue-agnostically approved for tumors with MSI-H [10] and ≥10 mt/mB TMB [11], and our patient met the criteria for both of these independent FDA approvals. Our patient’s tumor ARID1A mutation also supports a potential response to ICI [12]. Further, Lynch syndrome patients have been shown to have durable responses to ICI in various tumors, likely due to their germline MMR mutations [13].

There are potential reasons why our patient may not have responded to pembrolizumab. Even though the FDA approval is for ≥10 mt/mB TMB, patients with “high” TMB (>20 mt/mB) have been observed to respond better to ICI [14]. Although it is unclear why high TMB confers response to ICI, this is generally an accepted fact within the oncology community with a multitude of mechanistic hypotheses. Our patient classically would be referred to as “intermediate” TMB status (6-19 mt/mB), which has implications for lower ICI efficacy [15]. Additionally, her KRAS and ATM mutations would not have been targeted with pembrolizumab alone, but a logical combination of pembrolizumab with a KRAS inhibitor such as sotorasib and poly (ADP-ribose) polymerase (PARP) inhibitor like olaparib might have more comprehensively treated her cancer [16,17]. A combination regimen would have likely been ideal, as patients with CUP have been shown to have meaningful responses to drugs that target specific mutations observed on NGS [18,19]. Patients with CUP who are given drugs that target ≥50% of their mutations found on NGS have longer progression-free survival (95% confidence interval (CI), 0.11-0.64; hazard ratio (HR), 0.27; P=0.002), disease control rate (71% vs. 24%; P=0.003), and overall survival (95% CI, 0.17-1.16; HR, 0.45; P=0.09) [20].

## Conclusions

Although Lynch syndrome is the most common form of hereditary colorectal cancer and is associated with an increased incidence of endometrial cancer, there is much to be learned about the unique cancer manifestations that may arise in these patients. To our knowledge, this is the first report of Lynch syndrome-associated squamous cell CUP initially found in a retroperitoneal lymph node. Additional research is needed on emerging cancer presentations in Lynch syndrome patients, as these patients continue to live longer with precision medicine serving as a new frontier for the treatment of such novel cancers.
